# A Lecture on Sanitary Reform

**Published:** 1851-12

**Authors:** Lewis Rogers

**Affiliations:** Professor of Theraputics in the Medical Department of the University of Louisville; Louisville, Ky.


Art. III.—*A Lecture on Sanitary Reform.* By Lewis Rogers, M. D., Professor of Theraputics in the Medical Department of the University of Louisville.

Recognizing the wisdom and beneficence of the great author of nature, and in view of the vast amount of preventable sickness and mortality existent in the world as the result of removable causes, the illustrious Boerhave remarked that "the only disease natural to man was old age."

The period of three score years and ten is the common age which most human beings may reasonably expect to attain, unless prematurely cut off by accident or disease, the avoidable product of extrinsic influences.

The human constitution is by nature endowed with a vital force sufficent to sustain it, in comfort and vigor, up to this advanced and mature term of existence.

In what remarkable contrast to this natural longevity is the present average duration of human life as disclosed by the vital statistics of every part of the world in which sanitary inquiry has been even superficially prosecuted? A contrast appalling in its reality, a reproach to the vaunted civilization of the age, and strongly calculated to awaken the earnest consideration of every philanthropic mind.

The facts developed, in reference to human life, by the system of *registration of births, marriages, and deaths,* instituted within the present century, in Great Britain and on the Continent of Europe, and, to a limited extent, in some of the States and cities of this country, are at once startling and interesting; they disclose an astonishing subtraction from that common period of life immemorially allotted to the human race; a subtraction which has been silently but certainly devitalizing society since society began, robbing it largely of its strength and of its most fertile elements of happiness, none the less real and efficient, because the full and unceasing energy of its action had never been explored or appreciated.

The average age at death of all who die in London, is 26½ years, 32 per cent of all the deaths being of persons under 5 years of age, and the annual rate of mortality for the whole population being 1 in 39. Of the 49,089 persons who died in London in the year 1846, 22,275 died before they reached the 15th year of their age, and only 2,241 from that natural exhaustion of the vital force expressed by the term old age. In England the annual number of deaths from disease is about 300,000, while only 35,000 die from the mere decay and exhaustion of the frame by the progress of time. The average age at death in the county of Hereford is 38½ years, and in the county of Devon about 38 years, a range considerably higher than that of London; while in Birmingham the average age at death is less than 24 years, in Manchester less than 23 years, and in the great and flourishing commercial emporium of England, Liverpool, less than 21 years, figures very much below the reduced average of metropolitan longevity. The average age at death, for the whole of England, deduced from a large number of local averages, may be stated at 29 years, a duration of vitality considerably less than one-half of that which the human frame is supposed to be naturally capable of attaining under proper and wholesome influences.

"In Boston, from 1840 to 1845, 46.63 per cent. of all the deaths were those of persons under 5 years of age, and in some classes of the population, more than 62 per cent. were under that age; the average age of all that died in the same period was 21.43 years, and of the Catholic burials, 13.43 years only." In New York, in 1810 to 1820, it was 26.15, and in 1840 to 1843, it was

19.69. In Philadelphia, in 1810 to 1820, it was 26.25, and in 1840 to 1844, it was 22.01. In other towns and cities of this country, the average age at death is even lower than this, and is such as to justify the deduction of 20 years for the whole of the United States.

The wide departure which these statistics disclose from that advanced life which the normal human constitution is capable of reaching under favorable hygienic conditions, is one of the most remarkable and unexpected of the many curious developments of sanitary inquiry, and is sufficient to awaken an earnest scrutiny into the causes that impair, obstruct, and in many instances almost seem to annul the beneficent designs of the great architect of the universe. If man's organization be by nature such that it is competent to sustain itself in health, vigor, and usefulness, through the long period of 60 or 70 years, then the causes, through the agency of which, this organization, under the most favorable present circumstances, falters, decays, and ultimately perishes in the brief span of 20 or 25 years, must be powerful indeed, and should appeal, with the forcible argument of self-defence, to the prompt and searching inquiry of their unconscious victims.

In connection with this remarkable abbreviation of the natural term of human life incident to the vitiating influences of social aggregation, and to a certain extent, inseparable from this aggregation, it is worthy of serious remark and consideration, that the mere diminution of the number of years of life is, by no means, the only effect to be estimated ; for it must be obvious, that the same causes which so steadily shorten life must also be adequate to impair, very materially, the physical being, while it endures, and to impress upon it, disease and incapacity, various in kind and degree, but all tending to reduce the working or productive power of those who live and their capacity for the enjoyments so profusely afforded by the productions of nature and art. So very certainly is this true, that apart from all statistical inquiries on the subject, the very aspect of the inhabitants of towns, cities and States, where the rate of mortality is high and the average longevity low, is stamped with the distinguishable marks of a low grade of health, and serves at once to announce the malign influences to which they are exposed.

From a large number of unexceptionable facts again and again collected by statistical inquiries, it may be assumed as proved beyond all cavil or question, that mankind suffer an abridgment of the natural term of life equal often to one-half of that term, and in many cases exceeding it, and that associated with this abridgment and growing out of the same causes, there is a deterioration of health and an abatement of those energies that spring from a vigorous vital power, which detract very essentially from the enjoyment, happiness, and substance of a people.

It is a curious circumstance, it is indeed an anomaly very irrational and very difficult of satisfactory solution, that most persons bestow more care and attention upon all matters appertaining to themselves than they do to that one condition, *the condition of good health*, without which, all other things, however precious or costly, cease to confer pleasure or happiness, and become, in fact, utterly valueless. This remark is true in reference to men in all the ranks and conditions of life, and applies with scarcely less force and pertinency to the citizens of states and countries distinguished for refinement, and for the possession of all the comforts and luxuries which the manifold and cunning devices of art and science so abundantly furnish, than to less favored nations that have not yet gathered the fruits of an advanced civilization. Indeed, it would seem that the busy and elaborate inventions of art and science that multiply so wonderfully and so ingeniously the various productions adapted to the gratification of the wants and wishes of human sense, multiply, at the same time, the causes of disease, develope an increased susceptibility to the noxious influence of these causes, and engender a condition of health that more or less destroys all capacity for the enjoyment of the products of their labor. In proof of this, it is only necessary to survey the sanitary condition of the great commercial and manufacturing districts of the world; of those large towns and cities where wealth seeks productive investment, where population is densely crowded in quest of labor and the means of procuring the cheap necessaries of life, where industry ceaselessly plies her efforts, torturing inventive ingenuity and all the resources of science for new fields of enterprise, urged, on the one hand, by the sharp lash of want, and on the other, by the insatiable demands of avarice. Let the sanitary condition of towns and cities like these be fully investigated, and the inquiry will not be pushed very far before revelations will be made as to the health of the inhabitants which will appear too improbable to be true, and unsustained by authentic testimonials, would be rejected as false; before the anomalous proposition which we have advanced will be satisfactorily established, that the prolongation of human life and the preservation of human health, which should be objects of supreme and chiefest care, as constituting the fundamental elements of human happiness, are, in fact, very subordinate subjects of consideration, and are constantly sacrificed to the attainment of ends and purposes, which experience, often too late, discovers to be vain and profitless without them.

It is true that when men are positively sick, when the hand of disease and death has laid its grasp upon them and rendered them the subjects of immediate and pressing suffering, they are sufficiently prompt, for the most part, to seek the aid of curative medicine, and to demand the speedy interposition of professional assistance. It is equally true that their appeals for relief are often misguided, or directed towards sources whence safe and enlightened aid cannot possibly flow; this, however, is more the misfortune than the fault of the anxious sick, and is due, either to their want of proper discrimination and intelligence, or to the artful imposition of boastful and designing pretenders.

But the advocates of sanitary reform, though they may deplore this prevalent and pernicious error of the public mind, and earnestly suggest the means of its rectification, have nothing to allege on the score of public indifference to the remedial resources of the healing art, when present disease is developed; they have no plea to offer in behalf of the claims of curative medicine; on the contrary, a candid avowal of their opinions upon this subject, in view of the rates of mortality in many diseases and in many sections of the world, might repress, to some extent, the general obedience to these claims, and give support to the growing sentiment of some honest and philosophical observers that the world is physicked as well as "governed too much."

The great aim of sanitary reform, of the sanitary movement, as it is called, is, not to enforce the necessity of a larger share of attention to curative medication, but to awaken a more thorough investigation into the causes of disease, to suggest means by which these causes may be diminished, neutralized, or removed, to establish and sustain efficient and practicable measures, by which a large part of the sanitary evils every where prevalent, may be mitigated or prevented, and the necessity of curative interference greatly curtailed.

The science of medicine embraces within its legitimate scope, every thing which has any connection with the causation and cure of disease; all the more general and widely diffused, as well as the more limited and special causes; it is with the diversified effects of these upon the human frame that practical medicine has to deal, and for the palliation and cure of these, a large body of most enlightened and learned men, has, through all periods of the world's history, been zealously engaged. Diagnosis or the discrimination of disease, pathology or the history of morbid processes, and therapeutics or the application of remedial means for the restoration of diseased to healthy conditions, are the several leading departments of *curative* medicine. They have immediate cognizance of a great number of *effects*, due to causes, some of which are inscrutable in their intrinsic nature, though their habitudes and the circumstances under which they are developed may be known, and others are known and matters of familiar observation. It is the duty of every physician to make himself acquainted with the causes of disease as far as they can be explored, to study their modes of origin and action, and to suggest means adequate to their diminution and extinction; this, however, involves an incalculable amount of labor, and in its nature is so disconnected with the more urgent demands made upon his attention by the calls of positive sickness, that the claims of the latter have, for the most part, occupied the greater and more exclusive share of his notice, and the means for *preventing* disease have been, therefore, placed in a position subordinate to those for *curing* it.—This is not wholly the fault of the physician; he has but participated in that general indifference to the prevention of disease which has hitherto been so strangely prevalent in the public mind, and when urged by a sense of the importance of prevention, to recommend sanitary measures to public consideration, this apathy has confronted and baffled his philanthropic purposes. For this and other reasons, the attention heretofore given by the medical profession to the exploration of the causes of disease has been directed more by the influence which a knowledge of these causes may have upon the application of means for the relief of effects, than with the view of removing these causes and thus preventing their results.

Within a few years past, however, there has been springing up in many parts of the world a decided conviction that this was too limited a view of the subject, and that a knowledge of the *causes* of disease could be more successfully and efficiently applied to the *prevention* of their injurious effects, than to the cure. This conviction has found a place not only in the minds of members of the profession, but in those of statesmen, politicians, and of thinking men in all the professions and pursuits of life; it has grown into a general and public sentiment in many places where the fruits of its action have ripened sufficiently to be appreciated; the subject of sanitary reform, which is the offspring of this conviction, is becoming, and in fact, has become, one of the great and absorbing topics of discussion; it is esteemed one of the great levers for the amelioration of the condition of all classes, and more particularly the poorer classes of society; sanitary reform, sanitary science, the sanitary movement, and the sanitary question occupy a large space in the most influential literary, scientific, and political journals, and are engaging the efficient attention of rulers and the ruled, of the active and controlling spirits of the times. This is eminently true of England, France, and Germany; in our own country the subject is rapidly commending itself to public favor, and in some sections of it, has already enlisted a sufficient amount of interest to give very strong assurance of its ultimately commanding the warm support of all those most deeply concerned.

We have referred to the indifference manifested by mankind generally to the existence of disease or rather to the prevention of the causes of disease; the remarks made were probably too sweeping and general, and more applicable to modern times, than to the nations of antiquity; for it is unquestionably true that the institution of means for the preservation of health and the prevention of sickness has been, to a greater or less extent, an object of care with a few nations, even from an early period of history. Among the Jews, laws were enacted, and most religiously observed, the evident purposes of which were the sanitary welfare of the people; many of these laws were enforced as matters of religion, and were veiled under the sanctity which attaches to divine revelation, with the manifest object of more thoroughly commanding the rigid observance of their subjects; at the present time, these laws exercise a wholesome influence over this remarkable nation.

Among the Greeks and Romans, we have numerous evidences that they were not insensible to the value of those hygienic means which even at the present day are recognised as most important in the preservation of health and prevention of disease.

Among the former, great attention was paid to the physical education of both sexes, and the virtues of temperance and sobriety were fostered and enforced by the stern philosophy of their wise men, and the legal enactments of their rulers. In most of the Grecian cities, health officers were deemed essential to the public welfare, and the most distinguished citizens did not consider it beneath their dignity to serve in this humble but useful capacity; the hardy exercises of the gymnasium gave health, vigor, and activity to the body, whilst the philosophers, poets, and rhetoricians instructed the intellect and refined and humanised the heart by the creations of their genius.

Among the Romans, the public health was an object of especial and governmental care; the censors and ediles of this people were officers of great dignity and consequence; their most important functions were those of health officers; they supervised and controlled the construction of all buildings, both public and private, inspected all public stores of corn and other provisions, estimated the possessions of every citizen, registered his name, residence, quality, and the number of his children, corrected and reformed the manners of the people. The vital necessity for pure air was fully appreciated by the Romans, and all the means requisite to secure it were enforced in public and private buildings and throughout the city; traces of arrangements for this purpose still survive among the ruins of ancient Rome, as well as for the equally important object of preventing atmospheric pollution by theaccumulation of foul and offensive matter in a state of decomposition. The extensive system of drainage carried out by the mechanical skill and enterprise of the early Romans is among the most wonderful of the municipal achievements of that or any other people; vestiges of the sewers, even of the Tarquins still survive as remarkable monuments of architectural labor and strongly attest the sanitary sagacity of their authors; the bathing establishments of the Romans enjoy a classical reputation, and the supply of water for all other purposes was afforded at a cost that immeasurably surpasses that incurred for the boasted works of Cochituate and Croton.

Upon the destruction of Roman power and the consequent prostration of all the noble institutions to which it had long given the most generous support, and during the period of darkness which succeeded and extended over many successive centuries, every thing having reference to the preservation of human health was utterly neglected, and the causes of disease were permitted to have undisturbed and unresisted sway; the records of medicine for this protracted period are accordingly filled with the details of epidemics of the most malignant and fatal character, variously designated by names expressively significant of their virulence and power.

The sanitary movement of modern times, and of the present century, differs from that of antiquity in its more minute, thorough, and systematic character; it is founded upon a basis of statistical facts and philosophical principles; it recognises and leans upon, as its main pillar of strength and support, the invariable and immutable relation subsisting between cause and effect; it works and projects its measures by rules and laws carefully deduced from a large mass of accurate and reliable observations; it is a well designed scheme for the amelioration of the condition of human society in its most vital points, and gives assurance, in tones of earnest confidence, of its ability to promote the happiness of mankind, by the diminution or removal of those multiform causes that are now constantly weakening and obstructing the vital force, and abbreviating the natural term of life. To a system of measures so full of promise and so well founded in its pretensions, it is the duty of every good citizen to lend an impartial and attentive ear. To contribute to a proper understanding of the merits of the question, and to aid, as far as possible, the speedy attainment of results so desirable, we propose to analyse the character of sanitary science and sanitary reform; to lay open to view the gronnd on which on which they stand, and to present a brief abstract of their claims to public approbation.

The claims of sanitary science and sanitary reform derive their support chiefly from a few plain propositions, the truth of some of which is sufficiently obvious upon their mere enunciation, and of others has been sustained by the results of sanitary inquiry, in a manner and to an extent, that has excited the surprise of all who have given their attention to the subject; the facts and developments of sanitary statistics are indeed well calculated to excite the astonishment of those who have never been in the habit of looking beyond the mere surface of society.

A fundamental proposition of sanitary science is, that every effect springs from some adequate and commensurate cause; this is a law of nature as applicable to disease as to any thing else. No disease can arise, pervade a country, or a section of country, or attack a single individual, without being due to some atmospheric, local, or personal cause, the discovery and extirpation of which will, at once, arrest its further diffusion. The curative physician seeks for the causes of disease, as we have before observed, for the purpose of more thoroughly comprehending their modes of action upon the human economy; the sanitary reformer pushes his inquiries into the field of causation for the no less valuable and philanthropic purpose of staying the production of the morbid results which it is the province of the physician to palliate and cure; the efforts of the one are directed to the mitigation of ills which the more radical labors of the other aim to prevent.

The causes of disease are very numerous, and as diverse in character as they are numerous; they constitute indeed an extensive and almost boundless field for the labor of the sanitary reformer, and for the explorations of sanitary science; but it is one which the life and health of society require should be worked as thoroughly as possible, and the results appropriated to the purposes for which they are designed.

It may be thought that many of the causes of disease are so inscrutable in their origin and form as forever to elude the most patient and careful search for their discovery; this is, no doubt, the fact; if it be so, however, it is most proper to know it, as a mere matter of knowledge, and for the further purpose of discovering those circumstances, which, on the one hand, predispose to the easy access of such causes, and on the other, oppose barriers to their invasion. The cause of malignant cholera is unknown, and has resisted, and probably will forever resist, the most searching inquiries of the human mind; yet, no fact in medicine or hygiene is more conclusively established than that its malignity may be greatly mitigated or increased, by the accessory local circumstances resident in the place of its invasion. Opportunities for establishing this fact have been furnished in abundance by the two epidemics of cholera that have occurred within the present century; this pestilence has invariably prevailed, with its greatest virulence, in those places noted for the presence of atmospheric and personal conditions which all experience attests to be most powerful in vitiating the human system, and in engendering morbid predispositions. Though there are many seeming exceptions to this fact, though cholera sometimes spreads its terrors among the better classes of society, who are generally exempt from the pernicious influences tha favor its production, yet it is never so desolating in such quarters, and strict scrutiny will rarely fail to discover that the accessory elements of causation have stealthily passed the limits of their original or most common seats of production.

So of other epidemic and endemic affections. Their essential causes may be so occult as to baffle the most acute and skillful exploratory research, yet their habitudes may be discovered, the atmospheric, local, and personal conditions that increase or diminish the intensity of their action may be detected, and by the seasonable and proper application of a knowledge of their laws, their diffusion may be moderated and their violence greatly lessened or wholly annulled. This is especially true of the various forms of fever, from the prevalence of which in all parts of the world so large a portion of all the mortality arises, as to constitute them the leading tests of public health.

The causes of fever have ever been impenetrable subjects of mystery, and they yet constitute a "*terra incognita*" of the medical philosopher; but whilst the subtle nature of the febrile poisons has eluded detection, the accessory circumstances that favor their action are to some extent known, and may be successfully resisted. The illustrious Rush, seventy years ago, remarked that "to all natural evil, the author of nature had kindly prepared an antidote. Pestilential fevers furnish no exception to this remark. The means of preventing them are as much under the power of human reason and industry, as the means of preventing the evils of lightning and common fire."

The causes, of disease, both hidden and manifest, sanitary science seeks to unfold, and sanitary reform to advise and enforce the required measures of prevention. This constitutes the fundamental and motive principle of the sanitary movement, so justly denominated the "great idea of the age." It does not vainly essay to discover all of these causes, nor attempt to afford infallible measures for the prevention and eradication of all diseases; it knows that man, even under the most propitious circumstances, cannot always escape sickness, and that there are many conditions necessarily connected with the modes of life, business and pursuits of the social state that entail a number of unavoidable evils; yet upon the ground of first principles, upon reason and philosophy, and upon the indisputable evidence of statistical figures, which, when carefully collected and digested, may be considered as so many facts and truths, it does not hesitate to avow, in the language of the Massachusetts Sanitary Report, "that the conditions of perfect health, either public or personal, are seldom or never attained, though attainable; that the average of human life may be very much extended, and its physical power greatly augmented; that, in every year, thousands of lives are lost which might have been saved; that tens of thousands of cases of sickness occur which might have been prevented; that a vast amount of unnecessarily impaired health and physical debility exists among those not actually confined by sickness; that these preventable evils require an enormous expenditure and loss of money, and impose upon the people unnumbered and immeasurable calamities, pecuniary, social, physical, mental, and moral, which might be avoided; that means exist, within our reach, for their mitigation and removal; and that measures for prevention will effect infinitely more than remedies for the cure of disease." Sanitary science analysing and reducing to elementary forms the multifarious causes of disease, is enabled to present a few simple conditions as successful antagonists to their action; these simple conditions, the presence of which will generally suffice to ensure the enjoyment of good health, are pure air, cleanliness, good and sufficient food, and a moderate exercise of the mental and physical powers. This is no new discovery of sanitary science, for it is one, the truth of which has always been theoretically recognised, but it is one which this science attempts to display in its full strength and most impressive form, by an array of the facts and figures of statistical inquirers. By these facts and figures, the sanitary reformer hopes to reduce theory to efficient practice, to generate an earnest and operative conviction in the public mind, that just in proportion as these attainable conditions are enjoyed will be the general and individual health; that these influential agents are the measures of local salubrity, and that the difference between the healthfulness of different localities is to be attributed wholly to a difference in the degree of their presence, and that the privation and insufficient supply of these essential conditions of sound physical existence give rise annually to an enormous waste of health and life.

This enormous and preventable waste of human life, everywhere discoverable, is another proposition, the truth of which sanitary science proclaims, and upon which the friends of sanitary reform base the imperious necessity of the measures which they advocate. It may be inquired, what proof can be presented that this great waste of life really occurs? What proof can the sanitary reformer adduce of the correctness of the premises upon which he founds his claims to public attention? This question can be met with great promptness, and in a manner that cannot fail to be satisfactory to every mind competent to appreciate the plainest demonstration.

In several of the leading countries of Europe, and in some of the States and cities of this country, registration laws have been enacted and very fully carried into operation, by which annual record is accurately furnished, of the number of *births, marriages, and deaths;* a faithful record of these three most important of the personal and social incidents of human life supplies as sure an index of the sanitary condition of a country, as the returns of the exports, imports, and revenue furnish, of the production, consumption, and commerce of that country. By this system of registration, the relative healthfulness of different sections of a country, or of one country compared with another, can be ascertained with unerring certainty; the calculation is a simple arithmetical one, and can be relied on as perfectly, as figures in the adjustment of a business account.

In the reports of the Registrar-General, of Great Britain, as well as in those of several of the Health of Towns' Commissions, 2 per cent per annum, or 1 in 50, is fixed upon as a healthy and natural standard of mortality. It is assumed by the authors of these reports, after very careful deliberation, that 2 deaths per annum in every 100 of the population of Great Britain, is a reasonable and moderate standard, and as great a mortality as should or would occur under a proper system of sanitary regulations. The assumption of 2 per cent or 1 in every 50 of the population, per annum, is not a random and gratuitous one, but is educed from a careful and rigorous survey and comparison of the statistical districts of England and Wales; the deduction, from this survey, of 2 per cent, as a healthy and natural standard, is based upon the very reasonable hypothesis that the degree of health attained by the cities, towns, and rural districts of one section of the country is perfectly attainable by all, under proper sanitary measures. This standard is not derived exclusively from districts of country containing almost wholly an agricultural population, which is always more healthy than that of towns and cities, but from those districts which contain many densely populated cities where the causes of disease are more numerous and active, and a higher rate of mortality always prevails.

According to the fifth annual report of the Registrar-General, in 88 of the districts, the mortality was exactly 2 per cent, or 20 in 1000, or 1 in 50; in 52 districts it was 19 in the 1000, or 1 in 53; in 94 districts, it was 18 in 1000, or 1 in 56; in 78 districts, it was 17 in the 1000, or 1 in 59; in 39 districts it was 16 in the 1000, or 1 in 63; and in 15 districts as low as 15 in the 1000, or 1 in 67. According to this report, in a little less than two-thirds of the entire number of the registration districts, the mortality did not exceed 2 per cent, and in nearly one-half of that number fell short of that rate. These statistical records, which are entirely accurate and reliable, afford full justification for the assumption by the champions of sanitary reform, of 2 per cent, as a fair and natural standard of mortality. In the city of Birmingham, with a busy population of 140,000, the mortality is less than 2 per cent, or about 1 in 50.63; in London it is 1 in 39.10; in Sheffield, 1 in 29.28; in Liverpool, 1 in

34.92; in Leeds, 1 in 34.44; in Manchester, 1 in 39.93; in the whole of England, 1 in 45.8, or 2.18 per cent. The difference in the mortality of the different towns and cities of England, as here set forth, is striking indeed, and is altogether due, in the estimation of the Registrar-General, to causes which have no *necessary existence*, and might be obviated, to a great extent, by the enforcement of proper sanitary regulations. It is the opinion of this distinguished gentleman, and of many others engaged in the same philanthropic labors and observations, that no good and sufficient reason can be assigned why the towns and cities, in which the mortality annually ranges so high, should be more unhealthy than Birmingham and other cities equally fortunate; they unhesitatingly avow their conviction that, by wise sanitary measures it might be reduced, in all of them, to 2 per cent, or even less, and that thus a saving of life equal to the present excess of mortality above that rate might be effected, by which 50,000 valuable lives would be annually preserved. The statistics of Great Britain, fairly interpreted, fully sustain the proposition of the friends of sanitary reform, that there is a great and unnecessary waste of life every year; unnecessary because originating in causes that might be detected and removed.

If the standard of 2 per cent be a fair and natural one for Great Britain, it may be assumed with entire safety and propriety, as a standard of healthy and natural mortality in this country. There is nothing in the character of our climate, soil, or population, which can, of necessity, augment disease and death beyond the limits to which they prevail in Great Britain, and on the continent of Europe. We have, it is true, a greater diversity of climate than is to be found in Great Briiain, and there are certain morbid influences eonnected with the opening of a new country, which bring, in their train, a considerable amount of unavoidable disease; yet a large portion of this country has been settled sufficiently long to dissipate these causes, in a great measure, whilst the diminished density of our population, and the great cheapness and facility with which moderate labor can command all the necessaries and many of the luxuries of life, will more than neutralize, by the attendant improved sanitary condition of the people, the causes of sickness and death, which are entitled to be considered inevitable and unavoidable. Taking 2 per cent as a legitimate standard, for all sections of this country, as a state of mortality, which, under proper sanitary influences, need not be exceeded, it becomes interesting to inquire what is the actual existing mortality, and to what extent human life is wasted, in a country where life is so valuable, and the means of personal, social, and political happiness are so profusely enjoyed. Statistical inquiry has been prosecuted to too limited an extent to afford full information on this subject, in reference to much the larger part of the country, but sufficient is known, as regards a few of the most prominent and important points, to furnish a tolerably fair index of the probable condition of all the rest.

From the registration reports of Massachusetts, we derive very explicit information as to the waste of life in that State, and learn the fact, which, under less positive testimony, would scarcely have been credited, that, in the wealthy and flourishing city of Boston, distinguished for its good habits, its morals, and its learning, there is an annual waste of human life that rivals that of the most distempered districts of England. In Boston, according to the records, extending from 1840 to 1850, the mortality was 1 in 41, or nearly 2½ per cent, and for the last half of the decade, 1 in 34, or a fraction less than 3 per cent. This mortality exceeds that of the city of Birmingham, with its dense population, and the dust, smoke, and other nuisances connected with its unrivalled manufactories, and is very little less than that of Liverpool, which proverbially suffers the unenviable reputation of being the most unhealthy of English cities. According to the mortuary tables of these reports, if Boston had been under sanitary regulations, which would have kept its mortality down to the standard of 2 per cent, there would have been a saving of more than 1100 lives, annually, for the last ten years. For the year 1850, a very healthy year in the north, the mortality in Boston was 1 in 38, very nearly the same with that of the crowded city of London. The average annual mortality of Lowell, for 13 years, has been a fraction over 2 per cent, and for the whole of Massachusetts, rather more than 1½ per cent. The mortality of the city of New York, for 45 years, has been a fraction less than 3 per cent; of Philadelphia, for 34 years, a small excess over 2½ per cent; of Baltimore, for 14 years, 2½ per cent; of Charleston, for 27 years, rather more than 2½ per cent; of Savannah, for 8 years, rather more than 4 per cent; and, of New Orleans, for 4⅓ years, rather more than 8 per cent.

The statistics of these various cities, carefully collected, and altogether worthy of trust, demonstrate a mortality exceeding the natural standard of 2 per cent in every one of them, and in several of them, fearfully exceeding it; the precise extent of this excess may be accurately calculated for each of them, and may be made to assume a more startling appearance when set forth in figures; but this is unnecessary, for, presented as they are, they conclusively show an almost incredible waste of human life, due to causes which a searching investigation might detect, and a rational prudence might obviate. The difference between the mortality of Charleston, 2½ per cent, and of Savannah, 4 per cent, and of the latter city and New Orleans, 8 per cent, cannot possibly depend upon causes wholly beyond the control of sanitary measures; they do not differ so materially in local position, in local influences, in climate, in the character and pursuits of their population, as to justify this great and marked discrepancy in their rates of mortality. This excessive mortality, of some of the southern cities, is placed in bolder relief, by a comparison with that of other places. Dr. Pendleton, of Sparta, (Georgia,) derives, from the census returns of six counties of Middle Georgia, the very moderate per centage of 1.51, and further states that this is doubtless a fair indication for the whole of Middle Georgia, and will compare, favorably, with any other portion of the civilized world. What a terrible contrast does this present to the mortality of the principal city of that State, the 4 per cent of Savannah? The total population of New Orleans, for 4½ years, up to 1850, amounted to 466,384; during that period there were 37,785 deaths, which gives a mortality of 8.10 per cent, or 1 in every 12 of the inhabitants. New Orleans and La Fayette, with a population of less than 130,000, lost, by death, in 1850, a healthy year, 8,086; in the same year, Liverpool, the unhealthiest city in England, with a population of 370,000, three times that of New Orleans, lost, by death, only 10,123; the mortality of the former was rather less than 3 per cent, while that of the latter was 6¼ per cent; in every 1000 in New Orleans, 62 died, while only 27 in the 1000 died in Liverpool. If Liverpool be considered unhealthy, what are we to say of New Orleans?

But the picture of sanitary evils, everywhere existing, is not yet completed; it is not a picture of death alone which the sanitary reformer presents to the view; this is sufficiently dark and dreadful, but it is rendered far more sombre, by the numberless forms of disease and sickness, coincident with every death. It has been estimated, by careful sanitary statistis, that, for every death, there are 28 cases of sickness, and of course, *for every unnecessary*

*death, there are* 28 *cases of unnecessary sickness.* This is the estimate deduced by Dr. Lyon Playfair, from 12 years of observation of cases of disease and death, in the hospitals of Liverpool and Manchester; Mr. Shattuck makes the same estimate from 9 years observation in Boston. Mr. Farr estimates the number constantly sick as double the number dying in a year, which corresponds with the other estimates, allowing each case of sickness to continue 3 weeks and 5 days. Mr. Neison, forming his estimates from the returns of a large number of Benefit Societies, which, from the very nature and purposes of their organization, keep an accurate record of cases of sickness and death, gives 17 cases of sickness to 1 death; this is probably too low, from the fact that such societies usually exclude all persons below 21 and above 70 years of age, and do not note cases of mild disease and of less duration than a week. The editor of the Medico-Chirurgical Review thinks 20 cases of sickness to 1 death a very fair estimate, and Dr. Simonds adopts this ratio for the city of New Orleans; the records of the Louisville Marine Hospital show 14½ cases of sickness to 1 death, in the year 1850. Assuming all deaths over the standard of 2 per cent as unnecessary, and due to causes that might be detected and removed, and therefore, avoidable, what a startling account of unnecessary sickness and suffering could be rendered, for the various cities whose rate of mortality has been exhibited, and what a conclusive and irresistible argument is afforded to the advocate of sanitary reform? Boston has been proved to lose, unnecessarily, 1100 of her citizens, annually; upon the accepted basis of 28 to 1, she annually contains, within her limits, 30,000 persons unnecessarily sick. Mr. Shattuck, referring to the amount of unnecessary sickness in the State of Massachusetts, remarks, "that an abstract of the returns of deaths for 1849, has not been made, but, when it is made, we have no doubt that it will show an annual mortality as high as $2\frac{1}{4}$ per cent, or an excess in the whole of the State, of 6,000 unnecessary deaths, and of 12,000 years of unnecessary sickness." Dr. Simonds, speaking of the amount of sickness in New Orleans, says: "a mortality of 5 per cent is 20 cases of sickness to 1 death; 37,785 deaths have been before stated to have occurred here during $4\frac{1}{3}$ years preceding 1851; there were therefore, 755,700 cases of sickness. We shall suppose that the average duration is 2 weeks, presuming a greater prevalence of acute diseases; the number of days' sickness was, then, 10,579,800, equal to the constant sickness, during the entire period, of 6,687 persons, and equal to 28,985 years of sickness experienced during $4\frac{1}{3}$ years, by a population of less than 100,000, and equal to the entire lifetime of 1,159 persons, attaining to the average age at death generally attained in this community." It is calculated by Dr. Southwood Smith, that, in London there are annually 10,000 unnecessary deaths, and 250,000 cases of unnecessary sickness. This mode of presenting the subject may wear, to some, the appearance of exaggeration, but it cannot be considered too strong, or in the least tinctured with undue exaggeration, when it is recollected, that a large portion of this excessive sickness, a portion corresponding to the excess of mortality above 2 per cent, multiplied by 20, or even 28, is justly attributable to causes, the influence of which might have been withdrawn or prevented, by a well-devised plan of sanitary police.

Calculations of this kind might be indefinitely extended, and be made to show a state of things in reference to the sickness and mortality of most of the cities, towns, and even rural districts of this and every other country, which would appear incredible to all but those who have made this interesting subject a matter of special and searching inquiry. Few in this country have done this, and until within the present century, the great mass of mankind every where passed through life in ignorance of, or apathetic indifference to, the multiform causes which on every side and ceaselessly are distempering and vitiating that life, and contracting its duration within limits much more confined than those assigned to it by the unviolated laws of nature.

Whilst the startling facts of the sanitary statists have developed a condition of life scarcely known, and certainly never fully appreciated before, they, at the same time, are well calculated to elicit, and have efficiently elicited in many places, the important inquiry, whether it lies within the compass of human agency to institute measures adequate to the mitigation or amelioration, in any sensible degree, of the monstrous waste of health and life which these facts have conclusively demonstrated every where to exist? To this inquiry, the advocates of sanitary reform do not hesitate to give a prompt answer of affirmation, and they are prepared to do this, both from rational considerations and from the results of experience. The rational considerations upon which the opinion is based, that the present inferior sanitary condition is susceptible of very great improvements, by a well-digested, well-sustained and thorough system of hygienic measures, must be so obvious to every mind endowed with the power of tracing the connection between palpable causes and their manifest effects, that it is unnecessary to dwell upon them; the more sensible and tangible results of experience—results carefully ascertained and fully supported by the unerring test of figures—it may be both interesting and profitable to detail. The Massachusetts Sanitary Commissioners remark, that "sanitary improvements in England first began in the navy. It is observed in a sanitary report, that "so dreadful was once the condition of the Royal Navy, that in the year 1726, when Admiral Hosier sailed with seven ships of the line, to the West Indies, he buried his ships' companies twice, and died himself of a broken heart. Amongst the pictures then presented, as in Anson's voyages, 1740–'44, were those of deaths to the amount of eight or ten a day in a moderate ship's company; bodies sown up in hammocks and washing about the decks, for want of strength and spirit on the part of the miserable survivors to cast them overboard. Dr. Johnson, in the year 1778, thus describes a sea-life:—"As to the sailor, when you look down from the quarter-deck to the space below, you see the utmost extent of human misery; such crowding, such filth, such stench! A ship is a prison, with a chance of being drowned—it is worse, worse in every respect—worse air, worse food, worse company." In 1779, the proportion of deaths in the Royal Navy was 1 in 8 of the employed; in 1811, the proportion was 1 in 32 of the employed; and from 1830 to 1836, the average number of deaths annually, was 1 in 72 of the employed. And in this calculation the deaths from all sources are included—from wounds, drowning and all other external causes, as well as from disease. From the latter source the deaths were in proportion of 1 in 85 of the number employed annually."—These figures are eloquent beyond any words that can be employed. They excite, as they are fitted to excite, especially at first sight, our wonder. They ought, however, to occasion more of gratitude than astonishment, because the causes of the difference are not difficult to determine, and because almost all that appears in favor of recent times is due to the superior intelligence and humanity infused into the administration of the navy."

In this connection, and as an evidence of the perfect system of hygiene existing in our own gallant navy, I cannot forbear to allude to a recent event which has fixed the admiration of the world, and cannot fail to awaken in the bosom of every American, emotions of pride for the lofty virtue which conceived, and the unflinching heroism which executed the enterprise; I refer of course, to the expedition dispatched by Mr. Grinnell, under the auspices of the United States Government, in search of Sir John Franklin. This expedition has just returned, after an absence of eighteen months, in seas hitherto almost unknown and untravelled, "amid the frozen terrors of the Arctic ocean, helplessly drifting a thousand miles," uncheered by a ray of sun-light for eighty days, *without the loss of a single man.* The sanitary police of these vessels must have been perfect indeed, which could thus preserve their entire crew, amid the unparalelled hardships, sufferings and dangers of such a voyage.

"Equally good effects have followed the sanitary reforms in the British army. The mortality among the British troops at Hong Kong, in 1842, was at the rate of 19 per cent., or 190 in 1000; in 1843, it was 22 per cent., or 220 in 1000; and in 1844, it was 13½ per cent., or 135 in 1000. But during these years, the garrison was very badly accommodated; in 1845, their accommodation was greatly improved, and the mortality diminished to 8½ per cent., or 85 in 1000; and since that time, the troops having been lodged in what may be termed from their excellence, "model" barracks, their mortality at once dropped down to 2½ per cent., or 25 in 1000; a rate not much exceeding that of stations esteemed healthy. Since the adoption of the measure proposed by Dr. R. Jackson, of removing the troops stationed in the West Indies, to cantonments on the mountain ranges, the diminution in the rate of sickness and mortality has been such as to justify the assertion, that if this measure had been carried into effect at the time it was first urged by him, the lives of from 8,000 to 12,000 men would have been saved,—a sufficient lesson, one would think, to our authorities, not to *delay* the introduction of improvements which experienced medical officers concur in urgently recommending."

Macaulay, in his recent history, observes, in drawing a contrast between the present and the times of which he writes, "Some frightful diseases have been extirpated by science, and some have been banished by police. The term of human life has been lengthened over the whole kingdom, and especially in the towns. The year 1685 was not accounted sickly, yet in the year 1685 more than 1 in 23 of the inhabitants of the capital died. At present only 1 inhabitant of the capital in 40 dies annually. The difference in salubrity between London of the 19th century and the London of the 17th century is very far greater than the difference between London in an ordinary season and London in the cholera." Any one familiar with the mildest prevalence of epidemic cholera is prepared to appreciate the magnitude of this difference.

The terrible annual mortality of Liverpool, some years ago, was very justly attributable to the fact, that 40,000 of the citizens lived in cellars, and were overcrowded even in these miserable habitations. A resolution was formed and carried into effect "to amputate this morbid mass, by rendering cellars illegal habitations." By this measure and by others adapted to other special nuisances prevailing, Liverpool has, of late years, presented a striking and gratifying improvement in the health of her population; her rate of mortality has been lowered from 5.26 to 3.70 per cent, from 1 in 19 to 1 in 27.

Instances of the ameliorating influences of sanitary measures might be infinitely adduced, from the statistics of cholera, in every part of the world; none, however, can be more pertinent, or more conclusive of such influences, than the complete immunity enjoyed by the model-lodging houses and model prisons of London, many of which are situated in the most unhealthy districts, where the disease committed its greatest ravages.

The unquestionable facts now brought forward are certainly sufficient to gain for the subject of sanitary reform a calm and impartial consideration, if not to place it in the position of a settled question.

It would be impossible as well as inappropriate, in a general discussion of the sanitary question, to give the plans, in detail, which its advocates propose, for the hygienic improvement of the world. In a succinct manner and a few words, they may be stated to consist, in the institution of a thorough and pervading system of sanitary police, and the organization of boards of health, general and local, legally and scientifically competent to investigate the causes of disease; invested with power as far as human means are adequate, to neutralize, remove, and prevent these causes, and to advise and carry into execution all proper and reasonable measures for the promotion and preservation of personal and public health.

*The starting point* of a scheme of sanitary reform is the correct ascertainment of the precise sanitary condition of the place in which this reform is to be made; without information of this kind, the extent to which reform may be required cannot be known, and measures to effect it must be correspondingly uncertain, defective on the one hand, or transcending the limits of requirement on the other.

A complete estimate of the living population of a district, city, or town, and *an annual registration of the births, marriages, and deaths*, constitute the basis and essential elements of the knowledge required. From these, the sanitary condition of a people can be ascertained, with as much accuracy as the amount of revenue collected can be known from the books of the treasury department, or of exports and imports, from those of the custom house officer.

By the aid of statistics of this kind, the whole field of labor is clearly exposed to view, and those places distinguished for their salubrity may be made to furnish useful models for imitation, whilst those distinguished for their high rate of sickness and mortality, may invoke the prompt interposition of suitable measures of prevention.

The whole scheme of sanitary reform may be again briefly stated to have for its object, the ascertainment of the precise condition of the public health, the discovery of the causes which, on the one hand, promote, and on the other, diminish this health, the wide and universal diffusion of the former, and the total or attainable extirpation of the latter.

The machinery for the accomplishment of these several important and associated purposes, may seem to be, and in fact may be, complicated and difficult of adjustment; it may involve a large amount of labor and expense; it may require time and patience for its construction, and demand for its supervision and management, industry, skill, scientific attainment, and wisdom of a high order; as far, however, as sanitary reform has been attempted, requisitions of this kind have not been as great as the magnitude of the movement had led its friends to anticipate, and even if they had far transcended all reasonable anticipation, no adverse argument could thence find justification, for in no other cause can the ends to be attained so fully vindicate the means.

Every national, State, or municipal government is more or less costly and complicated; its executive, legislative, and judiciary departments demand the aid of talents and acquirements specially adapted to the nature of the duties to be performed; without an adequate remuneration, officers of a proper character cannot be commanded, the affairs of government suffer, and the interest of the people are sacrificed. A good government costs the outlay of a large amount of time, labor, and money, but then it returns to the governed an amount of benefit that more than compensates them, in the form of national importance and political happiness; no one feels oppressed by the burden of taxation, and every one contributes, with a willing hand, an appropriate share of the expense. So it is with all other institutions, whether of a social, commercial, or religious character; they are all, to some extent, complicated and expensive, yet they are all deemed necessary, and are bountifully sustained. So it would be with a thoroughly organized and efficient system of sanitary measures; and certainly, measures, which propose to improve the health, and preserve the lives of a people, are worthy to rank in the scale of usefulness and beneficence, upon a level with, if not superior to, all other measures designed to minister to the good of mankind. Without health and a reasonable longevity, it is impossible that a people can enjoy either social, personal, religious, or political prosperity.

The general view which has been presented of the great end and aim of the sanitary movement must address itself with convincing force to the minds of every prudent and rational people; but there are a number of special considerations connected with its economical, philanthropic, and moral influences, which cannot fail to become irresistible advocates in its favor.

The real strength and power of every State consist in the productive energy, industry, and labor of its citizens; that State which contains the largest population, of an age when the mental and physical powers are the most vigorous, a population capable of enduring, with ease and in health, sustained and protracted exertion, of producing largely beyond its consumption, must ever take precedence of others which are destitute of these qualities, no matter what may be the climate, fertility of soil, and other natural advantages of the latter; with a sterile soil and a rugged climate, and other physical obstacles to rapid growth, the faculties just referred to will vanquish them all, and ultimately build up a great and flourishing nation. Whatever diminishes or impairs these faculties, subtracts, in the ratio of diminution, from the wealth, prosperity, and importance of the State. A high and unnecessary rate of mortality, and of consequence, a corresponding high and unnecessary rate of sickness impair directly the productive power of a people, and are, consequently, chargeable with a pecuniary loss equivalent to the gains that would have accrued, under a system of sanitary regulations capable of restraining sickness and death within healthy and natural limits. Every healthy and productive citizen may be considered as so much capital profitably invested in business; the loss, by death, of such a citizen, is the loss of a certain amount of capital, sunk and gone forever; the loss of his labor is the loss of the interest upon that capital.

Every sick and unemployed citizen may be considered as so much idle capital, with a loss of interest equivalent to the loss of labor. Where the rate of mortality is high and the amount of positive sickness great, the entire population must, to some extent, feel the force of the morbid influences in operation, and feeble health and physical debility must every where prevail. A citizen thus enfeebled and distempered, not wholly incapacitated for work, but producing scarcely more than he consumes, is capital very badly invested, returning a very low rate of interest. The loss thus indirectly sustained by the diminution of the productive and industrial energies of a people is small, and individual in detail, but great and national in the aggregate.

In addition to this indirect source of pecuniary loss, there are several other more direct and positive losses worthy of consideration. Every case of sickness or death, whether necessary or avoidable, involves the outlay of no inconsiderable sum of money, to defray the cost of medicine and medical attendance, nurses and funerals. The expenditure of money for these purposes falls with special and often crushing severity, upon the humbler classes of society, who, from the privations and hardships incident to poverty, are sick oftener and longer than those who are placed in more prosperous circumstances. Money thus appropriated to meet the requirements of unnecessary sickness, whether occurring among the rich or the poor, may be considered an absolute loss, justly chargeable to defective sanitary measures.

A very considerable part of the pauperism, orphanage and widowhood of every country is immediately traceable to the premature death of relatives, who, under proper sanitary provisions, might have lived, and contributed to the comfortable sustenance of those dependent upon them. Lord Ashley, speaking of the pauperism of Great Britain, remarks that "at least one-third of the pauperism of the country arose from the defective sanitary condition of large multitudes of the people; and he had no hesitation in saying, upon the authority of experienced persons, that if the population of their great towns were placed under proper sanitary regulations, in less than ten years, the Poor Rates would be reduced three millions of pounds annually." An analysis of the recent documents and works of the sanitary statists of Great Britain proves, among other things, "that of the 43,000 cases of widowhood, and 112,000 cases of destitute orphanage relieved from the poor rates of England and Wales alone, the greater proportion of deaths of heads of families occurred from specified removeable causes." This is the fact every where, and the estimate would be a difficult and certainly a large one for this country, which would include all the money annually expended, in the support of our public charities, and lavishly scattered by the hand of private beneficence, for the relief of those made sick and rendered destitute, by causes which are cheaply removeable. Large as it is, however, it must be carried to the account of Profit and Loss, a wasting drain upon the public coffers, and a heavy burden upon private benevolence, for the at best imperfect mitigation of evils, which might be entirely prevented, at far less expense.

A large proportion of the crime of every country springs directly from its pauperism, and from the development of bad passions and bad principles, in early life, due to the want of parental authority and proper educational privileges. This crime, however produced, demands for its repression and punishment, the enactment and execution of the most rigorous laws. The police and judiciary departments of our cities and States are instituted at a very heavy cost to the public treasury, and unquestionably derive much of their business from the pauperism and depravity remotely engendered by defective sanitary measures.

The several economical propositions just presented are illustrated in a striking and imposing manner by the figures of the sanitary arithmetician. Dr. Southwood Smith says, "there are items of expense which may be reckoned to be incurred under the present system, or rather want of system;—direct attendance upon the sick; loss of what they would have earned; premature death of productive contributors to the national wealth; and expenses of premature funerals. Dr. Playfair estimates this loss for Manchester at nearly £1,000,000; Mr. Hawkesley calculates the loss for Nottingham at £300,000; Mr. Clay estimates the loss for Preston at £990,000; Mr. Coulthait takes the loss for Ashton-under-Lyne at £235,000; and Dr. Playfair considers the loss of London to be above £2,500,000; and that of England and Wales little short of £11,000,000; and of the United Kingdom £20,000,000, or nearly $100,000,000, and this an annual loss!"

Mr. Shattuck estimates the annual loss for Massachusetts, from these various sources, at $7,500,000; Dr. W. L. Sutton, of Georgetown, Kentucky, forming his calculations from the late census, estimates the annual loss of this State at $3,350,000. Dr. Simonds sets down the total loss of New Orleans, from these several items, during 4⅓ years, at the enormous sum of $45,437,700, "being an annual average loss of $10,485,623 to the city, and of nearly $105 to every individual in it."

There are some economical considerations connected with the sanitary movement, which are especially pertinent to this country. This nation, though great and powerful, may yet be considered in its infancy. We have, it is true, several large cities, but even these are the growth of a few years, and are destined to extend their limits far beyond what they are at present; most of our towns are small, though rapidly growing, and others are daily springing into existence. The whole face of the country is new, and may be improved after any fashion that its occupants may desire; never before, in the history of the world, was there presented a fairer field for human culture, or one more susceptible of adornment by the art and skill of an enlightened and cultivated people. Certainly, no where can a spot be found which offers so free a scope to the untrammelled action of the measures recommended by sanitary science; we have here few old prejudices and habits to combat; our boundless and inexhaustible territory obviates the necessity of an over-crowding of our population; few old and costly structures require to be removed to make place for such as may be deemed better adapted to the sanitary purposes for which they were devised; a fresh and virgin soil offers to scientific culture assurances of an abundant harvest.

The final aim of all sanitary measures is to afford to the people "an ample supply of pure air, pure water, light, sewerage and drainage, and extensive grounds for exercise." To afford these efficiently and successfully, in the laying out of our towns and cities, and in the construction of our public and private buildings, the precepts of sanitary experience and the principles of sanitary science should guide those to whose direction such matters are confided. In Great Britain, and on the continent of Europe, large sums of money have been squandered upon costly structures, which have proved utterly defective and inadequate to the hygienic purposes for which they were erected; not only have they failed to fulfill these purposes, but in many instances, they have manifestly increased the evils they were intended to remedy. In this country, all this may be avoided by a careful foresight, and an obedience to the suggestions of scientific skill; this, in the first instance, may seem to require an extravagant outlay of money, but in the end will prove a wise economy; "one broad principle may be safely enunciated in respect of sanitary economics;—that it costs more money to create disease than to prevent it; and that there is not a single structural arrangement chargeable with the production of disease which is not also in itself an extravagance."—Works constructed upon bad plans and incorrect principles require constant and costly repairs, which, without reference to their bad effect upon the public health, will be a source of endless and incalculable tax; when once properly constructed, they endure for a long time without change, perfectly fulfill their functions, diminish mortality, avert the physical suffering of sickness, and enhance the productive power of a community, in a degree that far more than reimburses the original cost.

The economical considerations just adverted to in reference to cities and States, are equally applicable to private capitalists; if they endeavor to economise by the erection of cheap buildings, destitute of proper ventilation, and with defective drainage and sewerage, neglecting the health and comfort of their occupants, though their dividends at first may be large, they will certainly be ultimately lessened by the impaired health and diminished productiveness of the immediate tenants, and of the community at large. A few individuals of wealth and philanthropy have made the experiment in England and on the continent, of erecting what are termed "model lodging-houses," for the use of the laboring classes; this improved kind of buildings has materially contributed to the comfort and healthfulness of the poor, and has yielded a return for the capital invested little short of the best; and this without exacting from tenants higher rents than are usually paid for very inferior houses.

There is a still further and final view of the sanitary movement, which is perhaps as interesting as any that can be presented. We have benevolent institutions spread all over the country for the protection, relief and cure of the blind, the deaf and the dumb, the idiotic and insane, orphans and widows; these institutions are fostered and sustained with a liberality and muuificence that indicate a large christian spirit and an inexhaustible philanthropy. But the sanitary movement presents a system of measures which reasonably and confidently proposes to discover the causes of the multiform maladies and misfortunes of mankind; which aims, in the language of Mr. Chadwick, "*to get at the antecedents, to mount to the sources*" of orphanage and widowhood, insanity and blindness, and all other forms of human suffering, and to apply the means of prevention at points where interference is not only more easy, but more radical and effective. Surely, the philanthropy which has ever been so prompt to alleviate, cannot falter or hesitate for a moment when called upon to prevent?

It has been well and truly said that "if it could be proved that filth and overcrowding, short supplies of water and aerial impurities are innocent of unnecessary sickness and premature death; if it could be shown that the structural arrangements of our cities and the nuisances with which they abound do not involve a sixpence of unnecessary expense, even then the sanitary question would hold its high ground among the great moral questions of the day." There seems to be an intimate and almost inseparable connection between physical and moral degradation; vice and filth are ever found in very close alliance; whatever debases the physical man is sure, sooner or later, to contaminate his moral nature; the favorite haunts of disease and death are the spots also distinguished as the hotbeds of crime and moral corruption in all their forms; "those who study the physical sciences and bring them to bear upon the health of man, tell us that if the noxious particles that rise from vitiated air were palpable to the sight, we should see them lowering in a dense black cloud above such haunts and rolling slowly on to corrupt the better portions of a town. But if the moral pestilence that rises with them, and in the eternal laws of violated nature is inseparable from them, could be made discernible too, how terrible the revelation! Then should we see depravity, impiety, drunkenness, theft, murder and a long train of nameless sins against the natural affections and repulsions of mankind, overhanging the devoted spots, and creeping on to blight the innocent and spread contagion among the pure. Then should we stand appalled to know, that where we generate disease to strike our children down, and entail itself on unborn generations, there also we breed, by the same certain process, infancy that knows no innocence, youth without modesty, or shame, maturity that is mature in nothing but in suffering and guilt, blasted old age that is a scandal on the form we bear."

It is too much the custom, perhaps, to look upon moral degradation as the cause of the physical; to consider the disease, want, filth and indecency of the abject outcasts of society as the results of their own uncontrolled passions and vices, and to view them with the stern eye of condemnation, rather than with the mild look of pity. The vice of intemperance is charged with the production of a large share of the want, disease and suffering of the lower classes; it may well be questioned, however, whether the absence of all domestic comfort in the miserable hovels of the poor, the exhaustion from the severe toil requisite to provide the scanty necessaries of life, the depressing and poisonous influences of vitiated air, do not engender a state of system that irresistibly seeks relief in the sedative stimulation of alcohol. The real and true relation between moral and physical degradation is, however, now beginning to be more correctly appreciated; under the influence of improved sanitary states of all classes of society, the important truth is being demonstrated that the moral as well as the physical condition of a people may be greatly ameliorated; ministers of the law as well as ministers of religion "are discovering that the scavenger and the architect are among their best allies," and by their arguments and eloquence, are urging the claims and speeding the progress of sanitary reform.

In this country, with a few unhappy exceptions, the monstrous moral and physical evils that so abound in most of the large cities of Europe, have fortunately as yet no existence; enough however exists plainly to foreshadow the future, and to enforce the wisdom and the necessity of a timely attention to the proper measures of prevention. If this be done, favored with all the blessings of sound health, sound morals, of civil and religious liberty, we shall, in future time, present a national spectacle without a rival in the world's history.

